# An Experimental Study on Static and Dynamic Strain Sensitivity of Embeddable Smart Concrete Sensors Doped with Carbon Nanotubes for SHM of Large Structures

**DOI:** 10.3390/s18030831

**Published:** 2018-03-09

**Authors:** Andrea Meoni, Antonella D’Alessandro, Austin Downey, Enrique García-Macías, Marco Rallini, A. Luigi Materazzi, Luigi Torre, Simon Laflamme, Rafael Castro-Triguero, Filippo Ubertini

**Affiliations:** 1 Department of Civil and Environmental Engineering, University of Perugia, Perugia 06125, Italy; andrea.meoni@unipg.it (A.M.); antonella.dalessandro@unipg.it (A.D.); marco.rallini@unipg.it (M.R.); annibale.materazzi@unipg.it (A.L.M.); luigi.torre@unipg.it (L.T.); 2 Department of Mechanical Engineering, Iowa State University, Ames, IA 50010, USA; adowney2@iastate.edu; 3 Department of Civil, Construction, and Environmental Engineering, Iowa State University, Ames, IA 50010, USA; laflamme@iastate.edu; 4 Department of Continuum Mechanics and Structural Analysis, School of Engineering, Universidad de Sevilla, Sevilla 41004, Spain; egarcia28@us.es; 5 Department of Electrical and Computer Engineering, Iowa State University, Ames, IA 50010, USA; 6 Department of Mechanics, Campus de Rabanales, University of Cordoba, Cordoba 14014, Spain; me1catrr@uco.es

**Keywords:** smart concrete sensors, self-sensing materials, structural health monitoring, strain sensitivity, carbon nanotubes, cement-based materials

## Abstract

The availability of new self-sensing cement-based strain sensors allows the development of dense sensor networks for Structural Health Monitoring (SHM) of reinforced concrete structures. These sensors are fabricated by doping cement-matrix mterials with conductive fillers, such as Multi Walled Carbon Nanotubes (MWCNTs), and can be embedded into structural elements made of reinforced concrete prior to casting. The strain sensing principle is based on the multifunctional composites outputting a measurable change in their electrical properties when subjected to a deformation. Previous work by the authors was devoted to material fabrication, modeling and applications in SHM. In this paper, we investigate the behavior of several sensors fabricated with and without aggregates and with different MWCNT contents. The strain sensitivity of the sensors, in terms of fractional change in electrical resistivity for unit strain, as well as their linearity are investigated through experimental testing under both quasi-static and sine-sweep dynamic uni-axial compressive loadings. Moreover, the responses of the sensors when subjected to destructive compressive tests are evaluated. Overall, the presented results contribute to improving the scientific knowledge on the behavior of smart concrete sensors and to furthering their understanding for SHM applications.

## 1. Introduction

Most current local monitoring systems (e.g., strain gauges, accelerometers, optical sensors, vibrating wire, etc.) provide limited assessment of the actual integrity of the monitored structure. Innovative solutions such as piezoelectric composites [1,2], micro-electromechanical systems (MEMs) [3], or fiber optic strain sensors [4], offer alternative monitoring solutions although hardly scalable to large-scale infrastructures without incurring high costs and utilizing complex signal processing algorithms [5,6]. Along these lines, recent advances in materials and nanotechnologies have permitted the development of novel multifunctional materials, which find a broad spectrum of applications in civil and aerospace engineering [7,8,9]. In particular, the superior electrical and mechanical properties of nanoengineered powders, such as Carbon NanoTubes (CNTs) and nanofibers, have resulted in several demonstrations of conductive cementitious materials with excellent sensing capabilities [10,11,12,13,14,15]. Such materials offer great promise for the monitoring of large-scale Reinforced Concrete (RC) structures. In virtue of the similarity of these composites and standard concrete, it is possible to develop mechanically robust sensors with vast potentials for conducting strain-based and vibration-based Structural Health Monitoring (SHM) [16,17,18]. Despite some limitations in the extensive manufacturing of composites doped with CNTs that have been reported in the literature, including the relatively high cost of the nanoparticles and complex fabrication processes related to their dispersion [19], the development of dense networks of embedded sensors offers a promising cost-efficient solution [20,21,22]. A network of small embedded sensors can monitor the host structure without interfering with its structural integrity. Nonetheless, some aspects concerning the electromechanical behavior of these sensors, including the static and dynamic response, as well as their behavior under large strains up to failure, still remain an open research issue.

Since the ’90s, the development of nanoengineered conductive particles has represented an important resource for the progress of engineering technologies [23,24]. Examples of application include smart nanocomposites, conductive coatings, nanodevices and nanoengineered materials [25,26,27,28,29,30,31]. Among other carbon-based fillers [15,32], CNTs have showed particularly promising capabilities. CNTs exhibit notable electrical and morphological characteristics suitable to produce electrically conductive networks throughout engineering materials such as concrete [11,33,34,35]. Furthermore, such particles have been reported to provide piezoresistive capability to insulating materials, leading to the creation of self-sensing materials with great potential in the field of SHM [36,37,38,39,40]. Self-sensing materials can be used to automatically assess the condition of a structural component through the analysis of data collected on-site, as well as to detect incipient damage and estimate prognosis with substantial economic benefits [41,42]. The self-sensing ability of CNT-reinforced cement-based materials or sensors is obtained through mapping variations in strain to variations in electrical characteristics of the material such as electrical resistivity or conductivity [43,44,45,46,47,48,49,50,51]. Research on CNT-based composites has primarily focused on dispersion strategies in different types of matrices [19,40,52], fabrication processes [53,54,55], and electromechanical response under quasi-static loads [56,57,58,59,60,61]. With regard to the application of nanocomposite cement-based sensors to SHM, it is worth noting the work by Han et al. [62], who investigated the use of MWCNT/cement composites as embedded strain sensors for traffic monitoring. Through vehicular loading testing, those authors reported good corresponding relationships between compressive stress and electrical response of the sensors. Saafi [63] developed CNT-reinforced cement-based sensors for crack detection applications in RC structures. Interfaced to a low-cost wireless communication system, small cubic CNT/cement sensors were embedded into 100 × 100 × 100 mm^3^ RC elements. Through three-point bending tests, Saafi’s results demonstrated sudden increases in the effective resistivity of the sensors when cracks initiate and start to propagate. Naeem et al. [64] analyzed the stress and crack sensing capabilities of MWCNT/cement composites subjected to flexural loadings. Additionally, those authors furthered the study by embedding MWCNT/cement sensors in different locations of reinforced mortar beams. A noteworthy contribution was made by Downey et al. [65], who proposed a novel biphasic DC measurement approach for use in the resistance measurement of self-sensing materials. Those authors demonstrated the applicability of the proposed approach for damage detection and localization using three different (40 × 40 × 160 mm^3^, 51 × 51 × 51 mm^3^ and 100 × 100 × 500 mm^3^) nanocomposite cement-based beams. The use of self-sensing cementitious materials for vibration-based monitoring was previously investigated by the authors [66,67], with particular attention to fabrication processes and electromechanical modelling of dynamic behaviors [51]. Research has demonstrated the potential of self-sensing cementitious materials at vibration-based SHM, but concluded that further studies are needed to better investigate signal quality and sensors’ response characteristics with varying amount of nanotubes and with or without aggregates.

This paper presents an experimental study on the behavior of a set of cement-based strain sensors fabricated with and without aggregates and with different MWCNT contents. The objective is to investigate the possibilities of CNT-reinforced cement-based sensors to be embedded in two key engineering materials, namely, cured cement paste and concrete, whereby concrete is the most used construction material worldwide, while cement paste is the matrix of any cement-based material. The investigation covers strain sensitivity and linearity of the sensors under both quasi-static and sine-sweep dynamic uni-axial compressive loadings. A study on the response of the sensors subjected to destructive tests carried out using a displacement controlled compression load completes the work. Overall, the presented results extend those obtained in previous research [19] regarding the effect of aggregates and different filler contents on the sensors’ behavior.

The paper is organized as follows. Section 2 describes the material properties and the preparation process of the samples. Also, the experimental methodology and the laboratory configuration for the electrical, electromechanical and destructive tests are illustrated. Section 3 presents the experimental results. Section 4 concludes the paper with a discussion of the obtained results.

## 2. Materials and Methods

### 2.1. Materials and Preparation Process of Samples

The cementitious sensors under investigation were fabricated with and without aggregates (cured cement paste and concrete matrices). The water/cement ratio was taken as 0.45 for all the admixtures. The cement was type 42.5, Pozzolanic. The mean diameter of sand particles was lower than 4 mm, while the mean diameter of medium gravel particles was between 4 and 8 mm. For neat concrete and nanofilled materials, a second-generation superplasticizer based on polycarboxylate ether polymers was introduced in a variable amount in order to obtain similar workability for all the admixtures. Table 1 and Table 2 list the different mix designs of cement pastes and concretes without filler (Table 1) and with different contents of MWCNTs (Table 2). The quantities refer to one cubic meter of produced material. In Table 2, Δ*V_P_*, Δ*V_M_* and Δ*V_C_* represent the incremental volume with respect to the reference cubic meter, composed of nanotubes and surfactant for composite cured cement paste and concrete, respectively, *n_%_* is the percentage of added filler with respect to the mass of the cement, and *C_p_*, *C_m_* and *C_c_* are the particular cement contents in the mixes of cement paste and concrete, respectively. The filler contents ranged from 0 to 1%, with step increments of 0.25%, and 1.5% for all specimens.

The carbon nano-fillers were MWCNTs, Arkema Graphistrength C100 [68]. They appear as black powder, with a carbon content greater than 90% in weight, and an apparent density of 50–150 kg/m^3^. The mean number of walls is between 5 and 15, with an outer mean diameter of 10–15 nm and a length of 0.1–10 µm. The surface area of the MWCNTs is approximately 100–250 m^2^/g. Their elastic modulus is greater than 1 TPa and their tensile strength is around 150 GPa.

**Figure 1:**
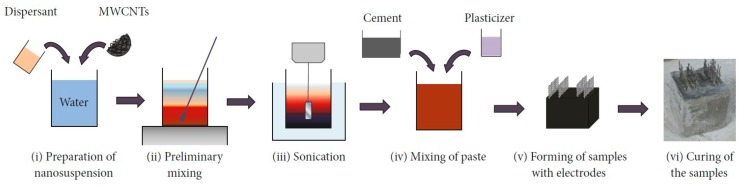
Preparation procedure of paste and concrete samples with carbon nanotubes.

Figure 1 describes the preparation procedure for the fabrication of paste and concrete cubes with MWCNTs. The process is divided into two subsequent steps. First, the carbon nanotubes were dispersed into water with a physical surfactant (i), mechanically mixed (ii) and sonicated (iii). Second, the suspension was added to cement and fine and coarse aggregates to achieve cement paste and concrete, respectively (iv). A plasticizer was added to obtain a similar workability of the fresh mixtures. The materials were casted into oiled molds, and the electrodes were embedded to a depth of about 40–45 mm (v). After 48 h, the samples were unmolded and cured for 28 days under laboratory conditions (vi). The fabricated samples are cubes of 5 cm sides in order to minimize the flexural efforts.

**Figure 2:**
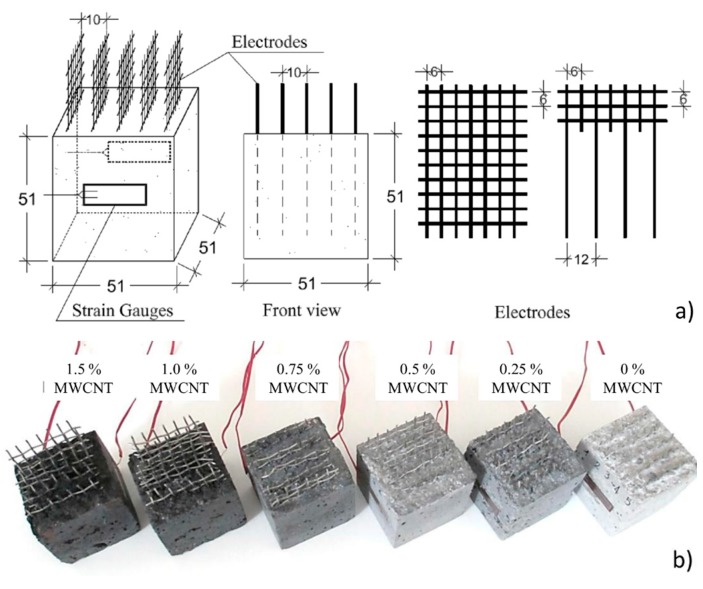
(**a**) Geometry of specimens and electrodes (dimensions are in mm); (**b**) Picture of samples with 1.5, 1.0, 0.75, 0.5, 0.25 and 0% (from left to right) MWCNTs.

Each cube was equipped with five embedded mesh stainless steel electrodes placed at a mutual distance of 10 mm. They were instrumented with two 20 mm long strain gauges installed on opposite sides. Figure 2a shows the geometry of the samples, the position of the electrodes and strain gauges, and the dimensions of a single electrode. For concrete specimens, the wire mesh was modified to be embedded at a distance of 12 mm (Figure 2a) so as to not interfere with coarse aggregates. Steel reinforcement bars were not included into the specimens and, therefore, the interest of the developed sensors focuses on compressive loadings. The applied loads during the electromechanical tests were uniaxial, perpendicular to the electrodes. The strain gauges were positioned at the center of the samples’ surfaces along the loading direction to measure the applied strain. The presence of different levels of MWCNTs resulted in different visual appearance for the samples. Figure 2b is a picture of samples with 1.5, 1.0, 0.75, 0.5, 0.25 and 0% MWCNTs contents. All the experiments were conducted under laboratory conditions at room temperature and humidity.

### 2.2. Electrical Tests

Electrical tests were conducted using DC current with a 4-probe method. A stabilized current was applied at two electrodes at a mutual distance of 30 mm, and the voltage, *V*(*t*), between the two adjacent electrodes, which were at a mutual distance of 10 mm, was measured for each sample. The data acquisition system used for acquiring measurements and providing the stabilized current was an NI PXIe-1073 device equipped with a high speed digital multimeter, model NI PXI-4071 and a current generator, model NI PXI-4130, capable of providing a four-quadrant ±20 V and ±2 A output on a single isolated channel. The electrical resistance of the specimens, evaluated after 6000 s of constantly applied current to achieve a stable level of polarization in the material, was obtained using Ohm’s law:

(1) $$R_{t}=\frac{V{\left(t\right)}_{t=t_{p}}}{I},$$

where *I* is the applied constant current, *V*(*t*) is the measured variations of voltage over time, and *t_p_* is the polarization time. The electrical conductivity, σ, was computed as follows:

(2) $$\sigma_{}={\left(R_{t=t_{p}}\cdot \frac{A}{d}\right)}^{-1}={\left(\frac{V_{t=t_{p}}}{I}\cdot \frac{A}{d}\right)}^{-1},$$

where *R* is the electrical resistance, *A* is the value of the section area of the sample, *d* is the distance between the electrodes.

### 2.3. Electromechanical Tests

**Figure 3:**
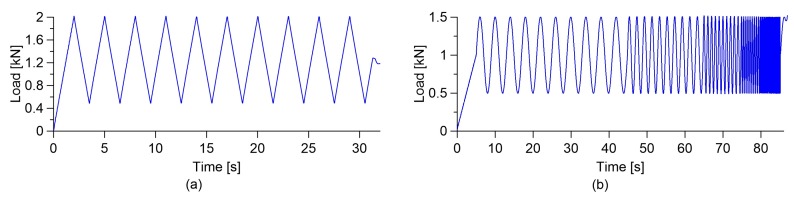
(**a**) Quasi-static uniaxial load; (**b**) Sine-weep dynamic uniaxial load.

The electromechanical tests for the assessment of the self-sensing capabilities were conducted using a servo-controlled pneumatic universal testing machine, model IPC Global UTM14P, with 196 kN of load capacity. The sensors were subjected to two different loading histories: the first one consisted of quasi-static loading-unloading cycles between 0.5 and 2 kN at a constant low speed (Figure 3a), while the second one consisted of a sine-sweep dynamic load with amplitude varying between 0.5 and 1.5 kN at increasing frequencies, from 0.25 Hz to 0.5, 1, 2, 4, and 6 Hz (Figure 3b). It is important to note that the selected frequency range contains the typical natural frequencies of large civil structures. The average compressive strain of the specimens was obtained by the two resistive strain gauges applied onto opposite faces, while the voltage variations over time were recorded through the data acquisition system. In a similar way to the electrical characterization tests, the 4-probe method was used for this test. The data acquisition system was an NI PXIe-1073, instrumented with a high speed digital multimeter, NI PXI-4071, a source measure unit, model NI PXI-4130, providing a stabilized voltage or current on a single isolated channel, and a data acquisition card, NI PXIe-4330, for strain gauge measurements.

The electrical sensitivity derives from several effects: the intrinsic resistance of carbon nanofillers and the cementitious matrix, the contact conduction among the nanotubes, and the tunneling and field emission conductions due to the nanosize dimensions of the nanotubes [22,61,69]. The relationship between the variation of electrical resistance, Δ*R*, and the axial strain, ε, can be assumed as follows (similar to the electrical strain gauges):

(3) $$\Delta R/R_{0}=-GF\cdot \varepsilon ,$$

where $R_{0}$ is the electrical resistance without load, and *GF* is the gauge factor of the material.

### 2.4. Destructive Tests

Destructive compression tests were performed by applying a uniaxial compression load under displacement control to the nanocomposite cement-based sensors using an electric-servo test machine, model Advantest 50-C7600 by Controls, equipped with a servo-hydraulic control unit model 50-C 9842. The axial displacement, applied with a constant speed of 2.0 µm/s, was measured through three linear variable differential transformers (LVDTs) connected to the test machine. The average displacement, *h*, was considered to obtain the axial strain, ε*,* following the equation:

(4) $$\varepsilon =\frac{l^{\prime }-l}{l}=\frac{(l-h)-l}{l},$$

where *l* is the original length and *l*′ is the final length of the specimen. Applied stress on the sample, $\sigma_{F}$, was calculated as follows:

(5) $$\sigma_{F}=F/A,$$

where *F* is the applied load measured through the load cell of the machine, and *A* is cross-section of the sample.

During the application of the compression load and up to failure of the specimens, electrical measurements were carried out with a 4-probe method using a high speed digital multimeter, model NI PXI-4071, and a DC current generator, model NI PXI-4130, both hosted into a chassis, model NI PXIe-1073, as illustrated in the case of electrical tests. This allowed testing of the sensing function within the whole range of deformation of the material.

## 3. Results

### 3.1. Percolation Threshold

**Figure 4:**
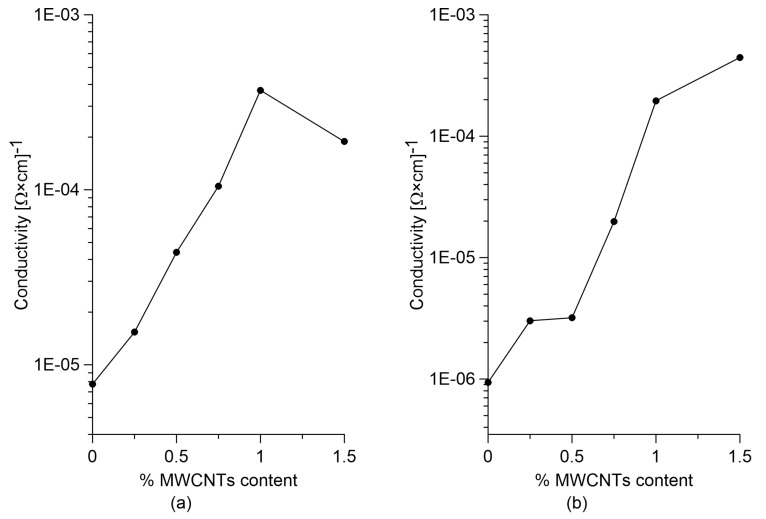
Electrical conductivity variation for different MWCNTs content in (**a**) cured paste samples; and (**b**) concrete samples.

Figure 4 plots the calculated electrical conductivity for cured paste and concrete specimens according to Equation (2). Results show that the percolation threshold is identifiable at 1% MWCNT content for paste specimens, and between 1% and 1.5% MWCNT content for composite concrete. The paste specimen with 1.5% weight content of nanotubes with respect to the weight of cement exhibits a reduction in electrical conductivity compared to the one with 1.0% MWCNT content. This can be attributed to a less homogeneous MWCNT dispersion because this specimen is the one containing the largest amount of nanotubes among those investigated. Another observation on the results is that paste samples without nanotubes are more conductive than plain concrete specimens, while after percolation, the two materials reach approximately the same conductivity. It follows that the addition of nanotubes has less effect on the variation of electrical conductivity in the case of cement paste compared to the case of concrete. In addition, the presence of aggregates in the concrete mixes results in a smaller effective volume to be filled by the MWCNTs particles, therefore making conductive chains more difficult to form in a cement and sand matrix relative to a cement-only matrix [70].

### 3.2. Linearity of the Sensors under Quasi-Static Compression Loads

**Figure 5:**
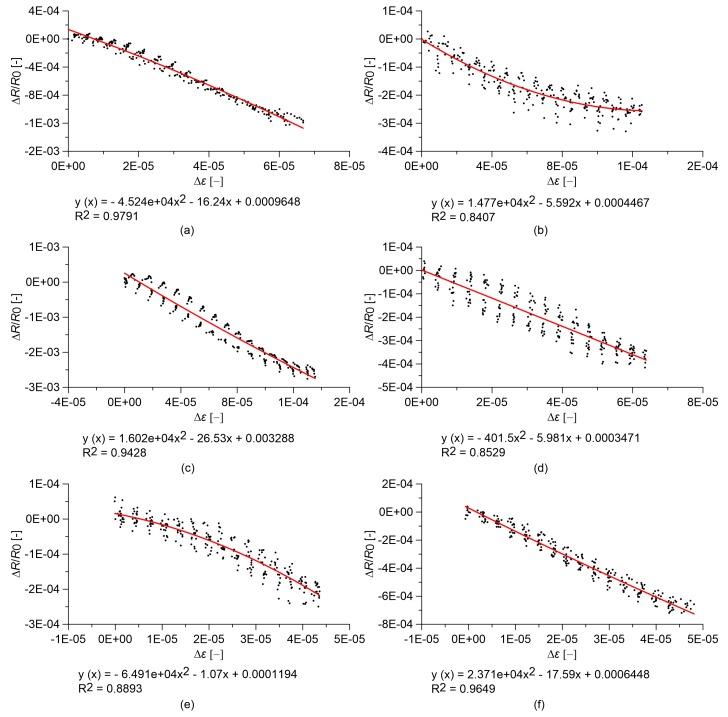
Relative change in electrical resistance versus applied strain of nanocomposite cement paste specimens under quasi-static compression loads. In the plots, *R*_0_ is the electrical resistance value with a preload of 0.5 kN, and equations of quadratic regression lines are reported: cured paste with (**a**) 0.00% MWCNTs; (**b**) 0.25% MWCNTs; (**c**) 0.50% MWCNTs; (**d**) 0.75% MWCNTs; (**e**) 1.00% MWCNTs; (**f**) 1.50% MWCNTs.

**Figure 6:**
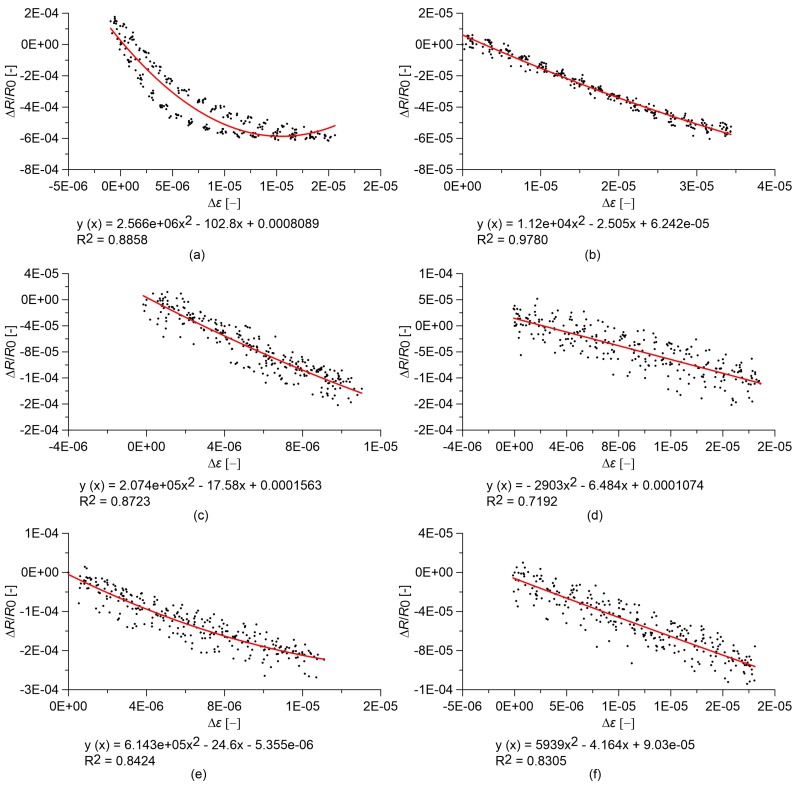
Relative change in electrical resistance versus applied strain of nanocomposite concrete specimens under quasi-static compression loads. In the plots, *R*_0_ is the electrical resistance value with a preload of 0.5 kN, and equations of quadratic regression lines are reported: concrete with (**a**) 0.00% MWCNTs; (**b**) 0.25% MWCNTs; (**c**) 0.50% MWCNTs; (**d**) 0.75% MWCNTs; (**e**) 1.00% MWCNTs; (**f**) 1.50% MWCNTs.

The strain sensing capability of the different specimens and their linearity are investigated through compressive tests with quasi-static loads. The plots in Figure 5 and Figure 6 report the relative change in electrical resistance versus the applied strain of cured nanocomposite paste and concrete samples, respectively. In both cases, the base materials without nanotubes exhibit clear strain sensitivity that is characterized by a significant non-linear relationship between the relative change in electrical resistivity and the applied deformation (Figure 5a and Figure 6a). This phenomenon is more evident in the concrete sample where the response of the base material also exhibits a hysteresis (Figure 6a). The addition of carbon nanotubes to the base materials regularizes such a strain-sensing response, making it more linear and reversible, up to a volume fraction of carbon nanotubes that is slightly below the percolation threshold (Figure 5b–d and Figure 6b–d). At percolation, the response becomes slightly non-linear for both materials (Figure 5e and Figure 6e), while the linearity seems to recover for a higher nanotube content (Figure 5f and Figure 6f).

### 3.3. Strain Sensitivity and Signal Quality

**Figure 7:**
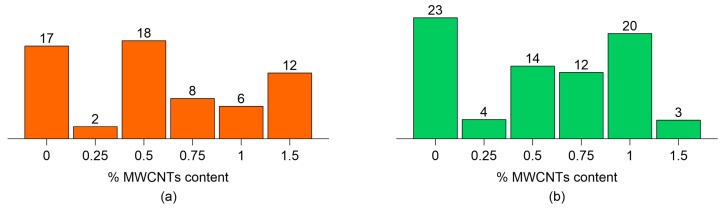
Gauge factor as a function of MWCNT content for (**a**) cured paste specimens; and (**b**) concrete specimens.

Figure 7 shows values of the gauge factor, *GF*, computed from the electromechanical tests depicted in Figure 5 and Figure 6, highlighting in particular the effect of a varying content of MWCNTs. As already observed above, both cured cement-paste and concrete specimens have demonstrated a clear strain-sensing capability though non-linear in nature, corresponding to relatively high *GF* values. Increasing the content of MWCNTs changes the conductive mechanisms and, therefore, the values of *GF*. Relatively large values of *GF* are obtained at 0.5% MWCNT contents for cement paste and 1.0% MWCNT for concrete specimens. The cement paste specimen with 1.5% MWCNTs seems to be an outlier, which could be associated with a less homogeneous MWCNT dispersion, as already commented on when introducing the percolation curves. These optimal amounts of MWCNTs resulting in the largest values of *GF* are close to the identified percolation thresholds, as also discussed in other literature works [56], confirming that the specimens exhibit an enhanced piezoresistive behavior near the percolation threshold. When the MWCNT content exceeds this optimal quantity, the nanotubes create a continuous network, and consequently an applied strain does not significantly modify the interactions between nanotubes that are already in contact. On the other hand, when the MWCNT content is lower than the optimal value, a reduction in *GF* is observed because the average distance between nanotubes is too large to allow the transfer of electrons from one nanotube to the other.

**Figure 8:**
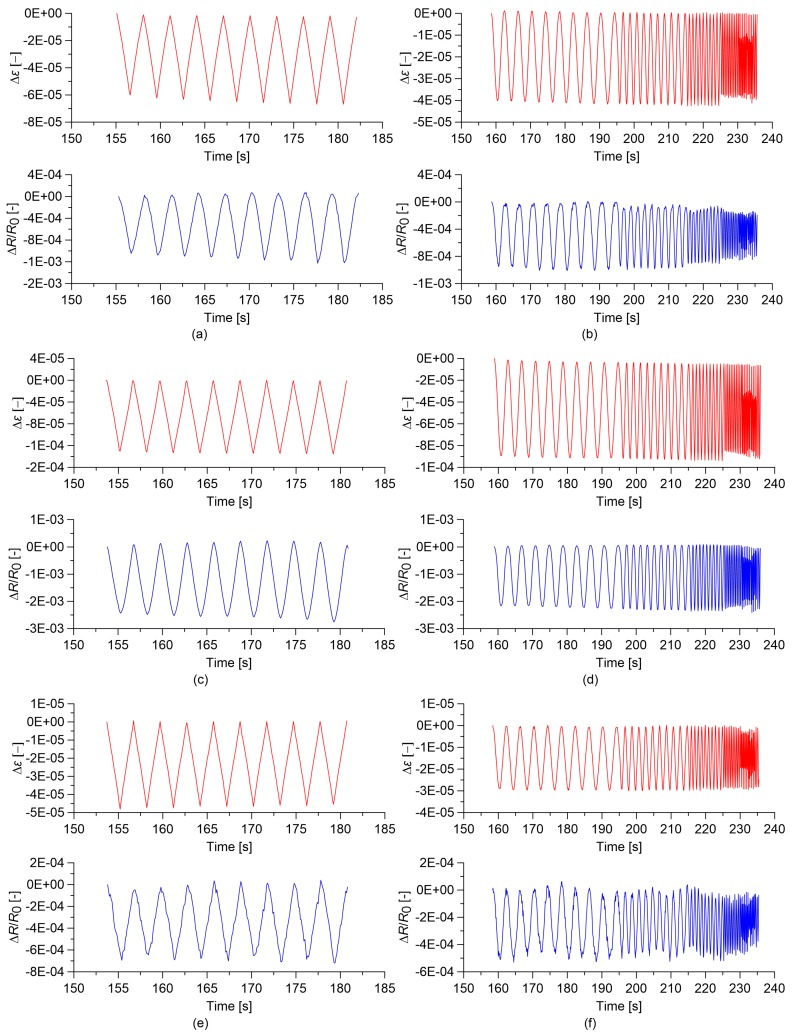
Time histories of the relative change in electrical resistance, Δ*R/R*_0_, and of the applied strain, Δ*ε*, obtained from the electromechanical tests. In the plots, *R*_0_ is the electrical resistance value with a preload of 0.5 kN: (**a**) Quasi-static load applied on cured paste with 0.00% MWCNTs;(**b**) Sine-sweep dynamic load applied on cured paste with 0.00% MWCNTs; (**c**) Quasi-static load applied on cured paste with 0.50% MWCNTs; (**d**) Sine-sweep dynamic load applied on cured paste with 0.50% MWCTs; (**e**) Quasi-static load applied on cured paste with 1.50% MWCNTs; (**f**) Sine-sweep dynamic load applied on cured paste with 1.50% MWCNTs.

**Figure 9:**
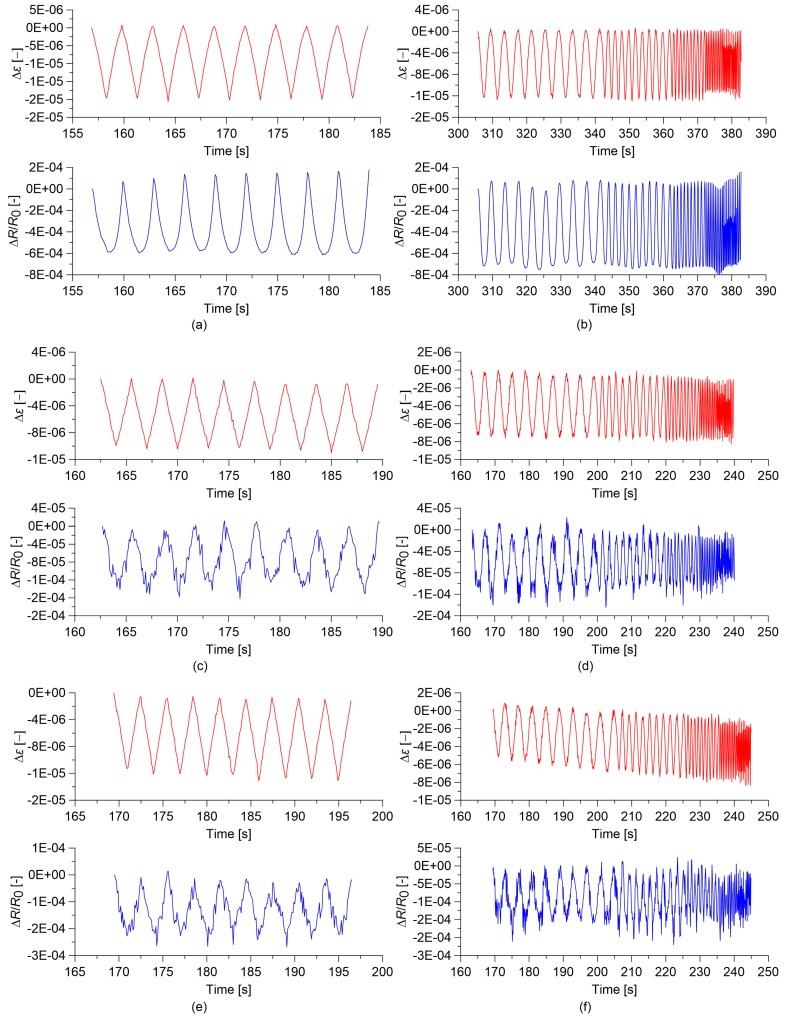
Time histories of the relative change in electrical resistance, Δ*R/R*_0_, and of the applied strain, Δ*ε*, obtained from the electromechanical tests. In the plots, *R*_0_ is the electrical resistance value with a preload of 0.5 kN: (**a**) Quasi-static load applied on concrete with 0.00% MWCTs; (**b**) Sine-sweep dynamic load applied on concrete with 0.00% MWCNTs; (**c**) Quasi-static load applied on concrete with 0.50% MWCNTs; (**d**) Sine-sweep dynamic load applied on concrete with 0.50% MWCNTs; (**e**) Quasi-static load applied on concrete with 1.00% MWCNTs; (**f**) Sine-sweep dynamic load applied on concrete with 1.00% MWCNTs.

Figure 8 and Figure 9 report the time histories of the relative change in electrical resistance, $\Delta R/R_{0}$, and of the applied strain, Δε, for cement paste and concrete specimens, considering the MWCNT contents corresponding to the optimal gauge factor values. It can be visually observed that concrete sensors exhibit noisier signals in comparison to cured cement paste sensors. Among cement paste ones, only the sensor containing 1.5% MWCNTs exhibits some signal distortions. It can be hypothesized that signal quality is highly affected by the quality of MWCNT dispersion, which is less homogenous in concrete in comparison to cement paste due to the presence of the aggregates and also among paste sensors in the case of the specimen with an MWCNT content of 1.5%, as already commented on above.

### 3.4. Slow and Fast Varying Response of the Sensors

**Figure 10:**
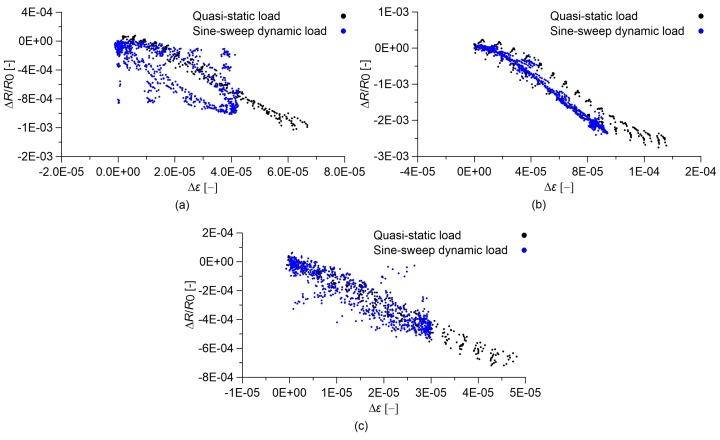
Relative change in electrical resistance versus applied strain of nanocomposite cured paste specimens. In the plots, *R*_0_ is the electrical resistance value with a preload of 0.5 kN. Comparison between results obtained under sine-sweep dynamic compression loads and those obtained under quasi-static compression loads: cured paste with (**a**) 0.00% MWCNTs; (**b**) 0.50% MWCNTs; (**c**) 1.50% MWCNTs.

**Figure 11:**
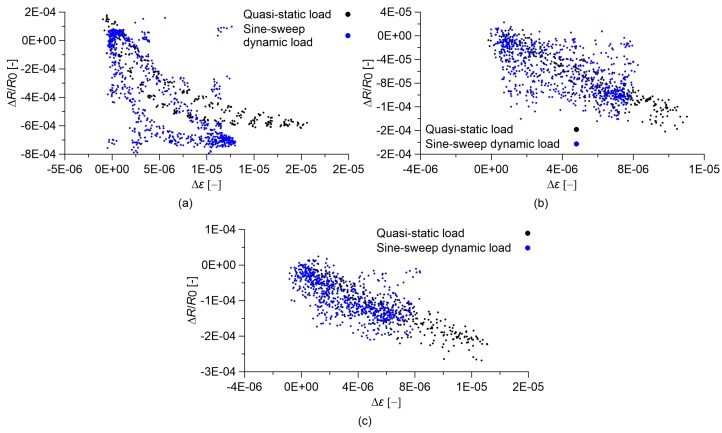
Relative change in electrical resistance versus applied strain of nanocomposite concrete specimens. In the plots, *R*_0_ is the electrical resistance value with a preload of 0.5 kN. Comparison between results obtained under sine-sweep dynamic compression loads and those obtained under quasi-static compression loads: concrete with (**a**) 0.00% MWCNTs; (**b**) 0.50% MWCNTs; (**c**) 1.00% MWCNTs.

Another aspect potentially affecting the strain sensing behavior of cement-based nanocomposite sensors is represented by strain velocity effects. To better understand this aspect, Figure 10 and Figure 11 show comparisons between the responses of the cement paste and concrete sensors under quasi-static and sine-sweep dynamic compression loads. Table 3 and Table 4 summarize the results obtained for cement paste and concrete specimens, respectively. In order to characterize the hysteretic behavior of the samples, the hysteresis area of the relative change in electrical resistance versus applied strain curves has been computed as the mean value of the areas enclosed by the loading/unloading cycles. These results show that the plain materials, besides behaving nonlinearly, also exhibit an important hysteretic behavior under the sine-sweep dynamic load, indicating a strong dependence of their electrical response under varying strain velocity. An increase in the MWCNT content has the effect of strongly reducing such a hysteretic response.

### 3.5. Response of the Sensors Subjected to Destructive Tests

**Figure 12:**
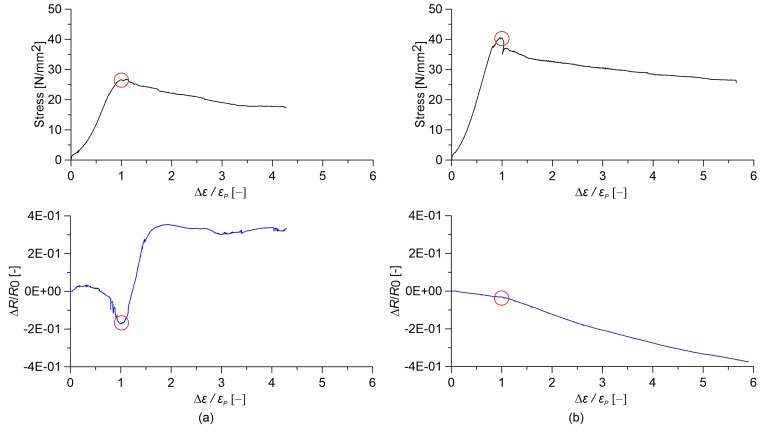
Comparison between stress and relative change in electrical resistance versus relative applied strain for paste specimens obtained from axial destructive tests (the circle indicates the peak stress point). Cured paste with (**a**) 0.00% MWCNTs and (**b**) 0.50% MWCNTs.

**Figure 13:**
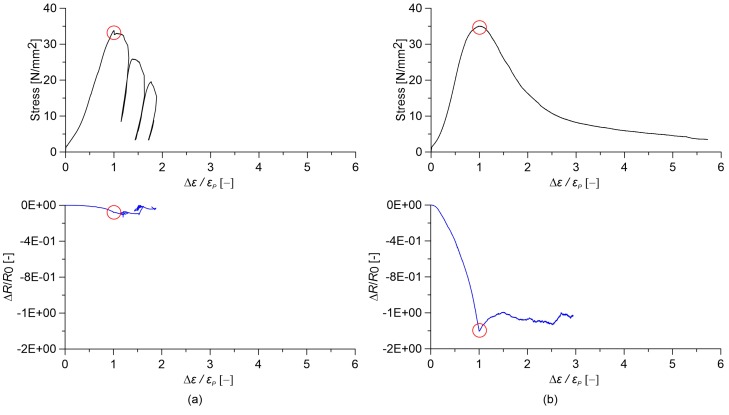
Comparison between stress and relative change in electrical resistance versus relative applied strain for concrete specimens obtained from destructive tests (the circle indicates the peak stress point). Concrete with (**a**) 0.00% MWCNTs and (**b**) 1.00% MWCNTs.

In this subsection, the electromechanical response of cured nanocomposite cement paste and concrete specimens under large deformations up to failure is investigated. Of interest are the sensing capabilities of these composites when cracking initiates and propagates. Figure 12 and Figure 13 show stress and relative change in electrical resistance versus axial strain obtained from destructive compression tests carried out on cement paste and concrete specimens, respectively. In order to avoid possible measurement errors in estimating small axial strain values using data outputted by LVDTs displacement sensors installed in the testing machine, strain values in *x*-axes of Figure 12 and Figure 13 are normalized by the strain at peak stress, denoted as $\varepsilon_{P}$. In both figures, plain samples are compared to the nanocomposite ones with the optimal gauge factor, corresponding to 0.5% and 1.0% MWCNT content for cement paste and concrete, respectively.

Plain cured cement paste exhibits a highly non-linear variation in the relative change in electrical resistance under the applied strain. In particular, when the strain reaches $\varepsilon_{P}$, a sudden increase in the electrical resistance of the material is observed due to the formation of compression cracks (Figure 12a). Afterwards, any sensing function is lost. On the other hand, cement paste doped with 0.5% MWCNTs exhibits a linear strain-sensing behavior, keeping the sensing capability at the ultimate strain. However, a slight change in the gauge factor is noted at peak stress (Figure 12b). Strain sensitivity is essentially lacking in the case of plain concrete specimens (Figure 13a), while the addition of nanotubes results in a high variation in the relative change in electrical resistance under strain, though slightly non-linear. Different from the case of cement paste, the sensing function is lost after reaching the maximum axial stress (Figure 13b). Note that the addition of MWCNTs is seen to increase the axial compressive strength of cement paste, as well as the ratio between the ultimate axial strain of concrete and the corresponding $\varepsilon_{P}$, therefore increasing its ductility.

## 4. Discussion

This paper has investigated the electrical properties and the strain sensitivity of two different cementitious materials with increasing complexity of internal structure, namely, cured Portland cement paste and concrete, doped with various contents of MWCNTs.

The electrical tests, conducted with a 4-probe DC measurement methods, resulted in the identification of percolation thresholds between 1.0 and 1.5% with respect to the mass of the cement. Cementitious sensors were subjected to electromechanical tests in order to investigate their strain-sensing capability.

Test results highlighted that both plain and nanocomposite cement-based materials exhibit strain sensing capabilities, whereby their relative change in electrical resistivity is affected by the applied strain. Plain cement paste and plain concrete, however, exhibit a significantly non-linear and hysteretic response. The addition of carbon nanotubes regularizes such a strain-sensing response, making it linear and reversible, up to a volume fraction of carbon nanotubes that is slightly below the percolation threshold. At percolation, the response becomes slightly non-linear, while the linearity seems to recover for a higher nanotube content.

When applying quasi-static and sine-sweep dynamic compression loads, cement paste sensors exhibit a better quality of signals compared to concrete sensors in terms of noise levels. Cement paste sensors exhibiting the most linear behavior under quasi-static loads also exhibit the least hysteretic response under sine-sweep dynamic loads. A similar trend for concrete sensors is not so apparent.

Destructive compression tests under controlled displacement conditions were performed in order to investigate the strain sensing capability under a large compressive strain up to the ultimate conditions of the materials. The results have highlighted the better strain sensing capability of the composite materials in comparison to plain ones. Cement paste doped with nanotubes held such a strain-sensing capability even after the peak compressive stress. An improvement in mechanical properties due to the introduction of carbon nanotubes is also evidenced for both paste and concrete specimens.

## 5. Conclusions

Results of the present research have confirmed that cement-based sensors doped with carbon nanotubes are promising for civil engineering applications, but the amount of nanotubes, the quality of their dispersion and the presence of aggregates are key factors that can highly affect their strain sensing behavior. Overall, it is concluded that nanocomposite cured cement paste sensors are more appropriate than concrete ones for strain sensing under quasi-static and sine-sweep loads because concrete samples exhibited a higher level of noise, conceivably due to lower homogeneous nanotube dispersion. The same result was obtained in destructive tests where nanocomposite cement paste has been found to be capable of maintaining the strain-sensing capabilities even after reaching the maximum compressive stress. Moreover, using cement paste sensors, a lower level of carbon nanotubes, in this research identified as approximately 0.5% with respect to the mass of cement, can be sufficient to achieve a good and linear strain sensitivity.

The presented results evidence the potential application of cement-based composites doped with carbon nanotubes as embedded sensors in key locations under compression of a full-scale structure (e.g., upper part of RC beams, columns, arches, etc.). Additional research questions need to be addressed by future studies, including the developments of cost-efficient dispersion and manufacturing processes, as well as the assessment of the influence of environmental conditions (temperature and humidity) on the electromechanical response of nanocomposite cement-based materials.

## Figures and Tables

**Table 1:** Mix design of cement paste and concrete without carbon fillers relative to one cubic meter of self-sensing materials.

Components	Cement Paste [kg/m^3^]	Concrete [kg/m^3^]
Cement 42.5 Pozzolanic	1277	524
Water	574	234
Surfactant	-	-
Sand (0–4 mm)	-	951
Medium Gravel (4–8 mm)	-	638
Superplasticizer	-	2.62
Water/Cement Ratio	0.45	0.45

**Table 2:** Mix design of cement paste and concrete with carbon fillers relative to one cubic meter of self-sensing materials.

Components	Cement Paste [kg/m^3^]	Concrete [kg/m^3^]
Cement 42.5 Pozzolanic	$C_{p}=1277\cdot \frac{1m^{3}}{1m^{3}+\Delta V_{PA}}$	$C_{c}=524\cdot \frac{1m^{3}}{1m^{3}+\Delta V_{CO}}$
Water	0.45 · *C_p_*	0.45 · *C_c_*
MWCNTs	*n*_%_ *· C_p_*	*n*_%_ *· C_c_*
Surfactant	*n*_%_ *· C_p_*	*n*_%_ *· C_c_*
Sand (0–4 mm)	-	1.8 · *C_c_*
Medium Gravel (4–8 mm)	-	1.22 · *C_c_*
Superplasticizer	variable	variable
Water/Cement Ratio	0.45	0.45

**Table 3:** Summary of the strain sensitivity analyses conducted on cured nanocomposite cement paste specimens, including Gauge Factors (GFs), and the hysteresis areas of the relative change in electrical resistance versus applied strain curves, considering both quasi-static and sine-sweep dynamic compression loads.

MWCNTs Content	*GF*	Hysteresis Area [µε] (Quasi-Static Loading)	Hysteresis Area [µε] (Sine-Sweep Loading)
0.00%	17	8.00 × 10^−4^	1.67 × 10^−2^
0.25%	2	4.09 × 10^−3^	3.17 × 10^−3^
0.50%	18	4.79 × 10^−2^	9.25 × 10^−3^
0.75%	8	4.89 × 10^−3^	2.46 × 10^−3^
1.00%	6	1.06 × 10^−3^	3.30 × 10^−4^
1.50%	12	2.67 × 10^−3^	2.52 × 10^−3^

**Table 4:** Summary of the strain sensitivity analyses conducted on nanocomposite concrete specimens, including Gauge Factors (GFs), and the hysteresis areas of the relative change in electrical resistance versus applied strain curves, considering both quasi-static and sine-sweep dynamic compression loads.

MWCNTs Content	*GF*	Hysteresis Area [µε] (Quasi-Static Loading)	Hysteresis Area [µε] (Sine-Sweep Loading)
0.00%	23	2.32 × 10^−3^	3.66 × 10^−3^
0.25%	4	3.00 × 10^−5^	3.70 × 10^−4^
0.50%	14	6.00 × 10^−5^	2.90 × 10^−4^
0.75%	12	3.50 × 10^−4^	4.60 × 10^−4^
1.00%	20	1.40 × 10^−4^	1.50 × 10^−4^
1.50%	3	2.60 × 10^−4^	4.00 × 10^−5^
